# Burnout among Healthcare Providers of COVID-19; a Systematic Review of Epidemiology and Recommendations 

**DOI:** 10.22037/aaem.v9i1.1004

**Published:** 2020-12-10

**Authors:** Mehrdad Sharifi, Ali Akbar Asadi-Pooya, Razieh Sadat Mousavi-Roknabadi

**Affiliations:** 1 Emergency Medicine Department, Faculty of Medicine, Shiraz University of Medical Sciences, Shiraz, Iran.; 2 Epilepsy Research Center, Shiraz University of Medical Sciences, Shiraz, Iran.; 3 Jefferson Comprehensive Epilepsy Center, Department of Neurology, Thomas Jefferson University, Philadelphia, PA, USA.

**Keywords:** Burnout, Professional, COVID-19, Coronavirus, Health policy, Workforce

## Abstract

**Introduction:**

In the current systematic review, we intended to systematically review the epidemiology of burnout and the strategies and recommendations to prevent or reduce it among healthcare providers (HCPs) of COVID-19 wards, so that policymakers can make more appropriate decisions.

**Methods:**

MEDLINE (accessed from PubMed), Science Direct, and Scopus electronic databases were systematically searched in English from December 01, 2019 to August 15, 2020, using MESH terms and related keywords. After reading the title and the abstract, unrelated studies were excluded. The full texts of the studies were evaluated by authors, independently, and the quality of the studies was determined. Then, the data were extracted and reported.

**Results:**

12 studies were included. Five studies investigated the risks factors associated with burnout; none could establish a causal relationship because of their methodology. No study examined any intervention to prevent or reduce burnout, and the provided recommendations were based on the authors' experiences and opinions. None of the studies followed up the participants, and all assessments were done according to the participants’ self-reporting and declaration. Assessing burnout in the HCPs working in the frontline wards was performed in four studies; others evaluated burnout among all HCPs working in the regular and frontline wards.

**Conclusion:**

Paying attention to the mental health issues, reducing the workload of HCPs through adjusting their work shifts, reducing job-related stressors, and creating a healthy work environment may prevent or reduce the burnout.

## Introduction

Burnout is a global health concern that affects physicians, nurses, and other healthcare providers (HCPs), and has been the focus of recent debates (1, 2). World Health Organization (WHO) recognized burnout as a syndrome and based on International Classification of Diseases (ICD)-11 it is defined as: ''Burnout is caused by chronic stress in the workplace which is not managed successfully and is characterized by three dimensions: 1) feeling of energy loss or fatigue; 2) increased mental distance from one's job or negative feelings or pessimism about the job; and 3) reduced professional effectiveness''. Burnout refers specifically to job-related issues and should not be used to describe experiences in other areas of life (3).

Burnout symptoms include frequent absences from work, a tendency to leave the profession, decreased self-esteem, and drug abuse, among others (4). Burnout is closely associated with reduced patient care level, increased incidence of medical errors, and lower patient safety (5-7). On the other hand, burnout may have negative effects on HCPs’ quality of life (6). Various studies have examined burnout in different health groups. A meta-analysis that was performed a decade ago, showed that 11% of nurses had experienced burnout worldwide (2). Many physicians may have similar experiences (1).

Since the beginning of the year 2020, the world has been experiencing an outbreak and a pandemic of coronavirus disease (COVID-19) that is caused by SARS-CoV2. By September 06, 2020, 216 countries were affected, nearly 27 million people were infected, and about 900,000 had died (8). Since the onset of the pandemic, HCPs, especially those working at emergency departments and departments that were specially devoted to treat COVID-19 patients, have faced a wide range of occupational stressors and a higher than usual workload; prolonged wearing of personal protective equipment (PPE), excessive heat caused by extra clothes, dehydration, poor nutrition, lack of enough sleep, and fatigue have predisposed HCPs to burnout (9). On the other hand, constant exposure to the suffering and death of patients and the constant need to sympathize with patients and their family members have caused extra mental health problems (10-13). With the onset of the pandemic, various studies have examined burnout among HCPs working in COVID-19 wards (11-15). In the current systematic review, we intended to systematically review the epidemiology of burnout and the strategies and recommendations to prevent or reduce it among HCPs of COVID-19 wards, so that policymakers can make more appropriate decisions.

**Data sources**

In this systematic review of the literature, we searched MEDLINE (accessed from PubMed), Science Direct, and Scopus electronic databases from December 01, 2019 to August 15, 2020, using MESH terms and the following keywords: (“COVID–19” OR “COVID19” OR “Corona” OR “Coronavirus” OR “SARS-CoV–2”) AND (burnout) AND ("Medical Staff" OR "Health Personnel"). Google Scholar and researchgate.net were also used to access other articles in English. To ensure literature saturation, the reference lists of the included studies or relevant reviews identified through the search were scanned.

**Study eligibility criteria**

We focused on the studies on the epidemiology of burnout and the strategies and recommendations to prevent or reduce it among HCPs. Articles were excluded if they were not relevant to the epidemiology of burnout, or to strategies and recommendations to prevent or reduce it, or were performed before the COVID-19 pandemic, through reading the title and the abstract. 

**Participants, and interventions**

The target population were all HCPs of COVID-19 wards (physicians, nurses, etc.). Moreover, we wanted to find which solutions or interventions are effective in preventing or reducing burnout among them.

**Study appraisal and synthesis methods**

**Figure 1:**
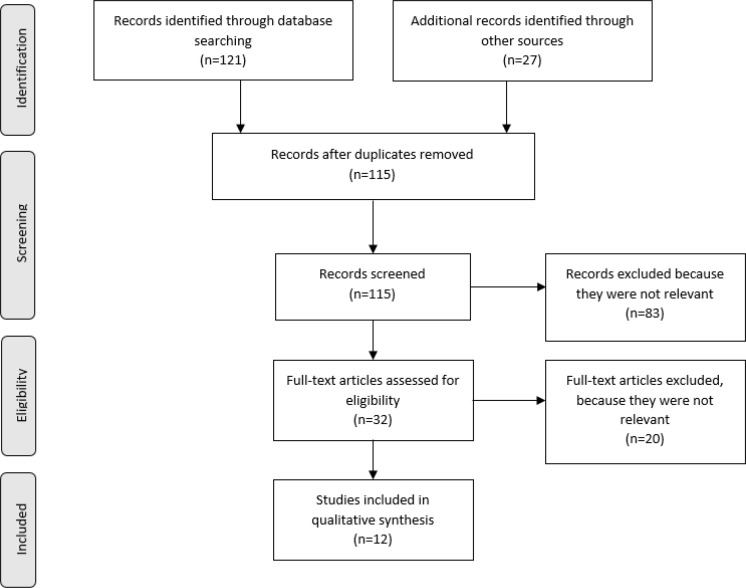
Preferred reporting items for systematic reviews and meta-analyses (PRISMA) flow diagram of the study

Then, full texts of the studies were evaluated by two authors (MS, RSM); they decided whether these met the inclusion criteria, independently. The quality of the studies was determined according to the American Academy of Neurology criteria for classification of evidence in causation studies (16). They resolved any disagreement through discussions, and finally the articles were selected based on consensus. Neither of the authors were blind to the journal titles or to the study authors or institutions. The following data were extracted from the included studies and recorded in a Microsoft Excel sheet, 2016: study authors, methods, main findings, and recommendations. This systematic review was reported according to the recommendations of the Preferred Reporting Items for Systematic Reviews and Meta Analyses (PRISMA) statement (17) (Figure 1). 

## Results

In total, 12 studies were included (9, 11, 12, 14, 15, 18-24). Table 1 shows the summary of these studies and their quality. Eleven studies were original articles with cross-sectional design; one study provided a conceptual paradigm for showing the relationship between acute stress disorder, posttraumatic stress disorder, and burnout (18).

Five studies investigated the risk factors associated with burnout (11, 18, 19, 23, 24); none could establish a causal relationship because of their methodology. No study examined any intervention to prevent or reduce burnout, and the provided recommendations were based on the authors' experiences and opinions. None of the studies followed up the participants, and all assessments were done according to the participants’ self-reporting and declaration. Eight studies used the Maslach Burnout Inventory (MBI) tool to evaluate the burnout (9, 11, 12, 14, 15, 19, 21-24); one used a questionnaire that was designed by the researchers (11); one used Stanford Professional Fulfillment Index (PFI) (17); and one study used a non-validated questionnaire (23). Web-based questionnaires through E-mail or social media were used in five studies (11, 15, 18, 19, 23). 

Assessing burnout in the HCPs working in the frontline wards was performed in four studies (9, 12, 14, 22); others evaluated burnout among all HCPs working in the regular and frontline wards (10-13, 15, 18-21, 23, 24). Four studies evaluated burnout among all HCPs, including physicians, nurses, technicians, paramedics, and other staff [1795 HCPs in all Taiwan hospitals (11); 1153 HCPs in frontline wards in Italy (12); 920 HCPs in Turkey (15); and 1422 HCPs in Spain (20)]. Three studies were conducted on nurses and physicians (14, 22, 23). In addition to the above-mentioned studies, we found 11 articles including opinions, editorials, or letters (6, 10, 13, 25-32). Table 2 shows the summary of these latter studies.

We categorized the related factors and the recommendations in five areas: 1. personal characteristics, 2. mental health status, 3. digital technologies, 4. workplace conditions and organizational behavior, and 5. the society (see also Table 3 ).

## Discussion

In this systematic review, twelve studies were found, which were about the epidemiology of burnout, or strategies and recommendations to prevent or reduce burnout among HCPs of COVID-19 wards. Most of the studies used the MBI tool to evaluate the burnout. MBI is one of the most common tools and the gold standard to measure burnout among staff, based on self-reporting using a Likert scale (33, 34). Our results showed that none of the studies were interventional, and none of them followed the participants. Although we categorized the related factors in 5 areas, most of the studies focused on the workplace conditions and organizational behavior as well as mental health status.

The results showed that burnout among HCPs working in the frontline wards was assessed in four studies; others evaluated burnout among all HCPs working in the regular and frontline wards. There are conflicting findings concerning the rate and epidemiology of burnout among HCPs working in COVID-19 wards. A study on 1,153 Italian healthcare professionals found that those who were directly involved with COVID-19 patients experienced higher levels of job-related stress, somatic symptoms, and burnout. Burnout, particularly emotional fatigue and depersonalization, was directly associated with the experience of at least one somatic symptom (such as changes in eating habits, difficulty sleeping, and muscle tension) during the past 4 weeks (12). In another study, 40.3% of the HCPs of COVID-19 wards, particularly nurses (45%) and physicians (31%), experienced burnout (11). A study from Turkey found that burnout rate was higher among the staff of emergency departments, ambulances, and intensive care units (ICUs), who were in the first line of combat against COVID-19 (15). One study reported that trainees who were exposed to COVID-19 patients had higher rates of burnout compared to those in the non-exposed group (18). Another survey found that nurses had experienced the following: 60.5% emotional fatigue, 42.3% depersonalization, and 60.6% decreased self-adequacy (19).

In contrast to the above-mentioned studies, one study reported that those working in COVID-19 wards had significantly lower levels of burnout compared with physicians and nurses working at other wards; the former HCPs felt higher levels of control over their work, they were more aware of the preventative policies and procedures, and were supported by the healthcare system. Furthermore, the staff working at the COVID-19 wards felt more valued (14). Another study on first-line residents (e.g., emergency medicine, radiology, and ICU), showed that 76% of them had burnout, which was lower compared to that among residents at other wards (rate of 86%) (9). Further studies are needed to clarify the frequency of burnout among HCPs working under different circumstances during the COVID-19 pandemic.

Various studies have mentioned several associated factors for burnout. HCPs may experience higher levels of workload, are engaged with strict organizational regulations, have less time to deal with their job challenges, and the knowledge in the field is continuously evolving (35). Furthermore, during the COVID-19 pandemic, an uncertain prognosis of patients; lack of enough medical resources for diagnosis, treatment, and prevention; problems related to protecting healthcare providers from getting infected due to inadequacy of PPE; rapid change in public health-related policies; decreased income and economic recession; and conflicting information announced by officials have been major stressors that certainly may increase the risk of burnout (36).

Health managers and policymakers' awareness of burnout is important in prevention and appropriately addressing it. A meta-analysis (2018) showed that resilience reduces burnout (37). Therefore, during the COVID-19 pandemic, it is necessary to recognize the factors associated with burnout and also identify the ways to deal with them. Different studies have suggested various methods to prevent or reduce burnout. These methods may be divided into two categories: individual methods and organizational (system-based) approaches (9, 13, 14, 19, 24, 26). 

Some studies reported that women have higher levels of emotional fatigue than their male counterparts (12, 26). Also, men may experience fewer somatic symptoms (12). One study showed that being a woman is a risk factor for experiencing burnout among HCPs working in acute critical care division (11). On the other hand, another study reported that burnout was not associated with gender (37). It was also reported that burnout was more common among HCPs who had a child or a family member older than 65 years or with a chronic illness, due to fear of transmitting the infection (15). 

On the other hand, maintaining physical and emotional hygiene is an effective strategy to reduce burnout. Happiness, regular exercise, drinking water, and having a good rest may increase the immunity and keep the person away from the disease (11, 24, 25, 38) (11, 25, 38). Therefore, simple measures such as providing a resting facility and the possibility of taking a shower at the workplace may be effective (38, 39). Interaction with family members and loved ones (40) and social support by the family (19, 29, 30) are other effective measures in reducing burnout. 

One of the important factors associated with burnout is the mental health status (33). Burnout is a multi-dimensional response to job stressors. These stressors may be physiological, emotional, or interpersonal (41). Burnout may lead to increased rates of psychological problems, suicide, and substance use among HCPs (20). Obligation to provide selfless service to the community may lead to neglecting their own physical, mental, social, and emotional health among HCPs (10). Improving work schedules, promoting self-management, teaching physical, mental, and emotional self-care, and starting mindfulness-based stress control activities are among the effective techniques to prevent or reduce burnout (10, 13, 28). Providing counseling and support systems, as well as holding support meetings for COVID-19 treatment teams are other effective interventions (12, 26, 27, 35). HCPs should be heard, protected, prepared, and supported by their organizations. 

Digital technologies may be a causative factor for burnout and also may be used to reduce burnout. In recent years, the role of digital technologies in providing health services has expanded. During the COVID-19 pandemic, registry systems and electronic health record (EHR) systems have been used widely (32). These systems should serve physicians and HCPs, but at the same time, EHR systems monitor physicians' performance and their qualifications. Therefore, instead of spending time to provide health services to patients, physicians have to enter the data into the EHR; as a result, they spend more time at the hospital and stay away from their families; these may cause burnout (6). 

On the other hand, digital technologies, such as mobile applications and social media, can be used to provide mental health services and increase the empowerment of HCPs (32). Talking about concerns with colleagues and friends, which can be achieved through web-based social media, is an appropriate way to reduce the stress (9). Also, the use of digital communication platforms, such as WhatsApp, allows physicians to access each other more easily, share information, and have immediate access to valid and updated information. 

Burnout is often influenced by organizational behaviors. Changing the behaviors that may cause burnout and adopting healthier behaviors is essential. This can only happen if there are organizational interests to meet these challenges (10). A meta-analysis showed that workplace interventions were directly associated with a reduction in the burnout scores (35). Therefore, along with other individual measures, interventions to improve the workplace and organizational environment have significant effects on promoting work culture and relieving workplace stress (9, 13, 20, 24, 26).

The number of work experience years, the number of working hours per week, more night shifts per week, the frequency of working over the weekends, having a coworker who is suspected or has a confirmed diagnosis of COVID-19, and the number of staff members in each team may be associated with burnout (24, 42). Organizational strategies to create a capable environment to reduce burnout could include the following interventions: improving workflow management, organizing services with an emphasis on reducing workload, improving communication skills, arranging discussion meetings, increasing interoperability, providing the opportunity for having adequate rest and exercise, holding workshops on coping skills, decreasing the clinical demand via schedule changes, and increasing teamwork (19, 25, 31, 32). Developing clear and up-to-date guidelines and protocols for different situations, as well as practical training about protective interventions are among interventions that may increase the sense of safety, assurance, and control (9, 24, 26, 31). 

Finally, the WHO has stated that an imbalance between effort and reward may lead to feelings of injustice or incompetence, which in turn leads to the feeling of anger that may be directed against the supervisor or co-worker (43). To reduce burnout, there should a balance between giving and taking, stress and relaxation, and work and home (44).

Burnout may be associated with social support outside the family (19). Social interactions of HCPs are effective in reducing burnout (29, 30). Wearing face protection equipment may lead to deterioration of the interpersonal relations and interactions due to difficulty in face recognition. To solve this problem, it was recommended to install photos of the staff on their clothes (9).

**Table 1:** Articles included in this systematic review and their main findings

**Author**	**Methods**	**Main findings**	**Recommendations**	**Level of evidence**
Dimitriu MC, et al. (2020)^(9)^	Cross-sectional study to compare the frequencies of burnout syndrome among 50 medical residents working in the frontline wards (30 emergency, 10 radiology and 10 intensive care unit) and 50 medical residents working in normal hospital wards (25 surgery, 15 obstetrics and gynecology, 10 orthopedics) during the COVID-19 pandemic.	Burnout was significantly more frequent in medical residents in normal wards (86%) compared to medical residents working in frontline (76%). Effective measures must be taken at the institutional and individual levels. Balance between giving and taking, stress and relaxation, and work and home. The shift program must be planned in a way that respects the epidemiological timing (incubation period or quarantine time). Periods of rest and relaxation must be observed. Practical training sessions should be held on the use of PPE.		III
Sung CW (2020)^(11)^	Cross-sectional study to evaluate burnout, anxiety symptoms, acute stress disorder, and health literacy and promotion among 1,795 HCPs in Taiwan hospitals during the COVID-19 pandemic.	45% of nurses and 31% of physicians suffered from burnout. Burnout was: 30% higher in HCPs who worked in the acute critical care division (ACC). 87% higher in HCPs who had taken care of suspected or confirmed cases. 9 times higher in HCPs with depressive disorder. 24% lower in HCPs who had higher health literacy and engagement in promotion activities. HCPs in the ACC had higher risk of burnout if they: Were Female Were a physician or nurse Had no previous experience with SARS or MERS Had severe anxiety	Avoiding excessive and unnecessary preventative measures. Ordinary or modest self-protection measures rather than an aggressive change of daily habits may be a better strategy. Health literacy and health promotion behavior. Being joyful, exercising, drinking water, and having a good sleep.	III
Barello S, et al. (2020)^(12)^	Cross-sectional study to describe the levels of burnout and physical symptoms of 1,153 Italian HCPs in frontline directly involved in the care of patients with COVID-19.	>33% had high emotional exhaustion scores. 25% had high levels of depersonalization. 15% had low levels of personal satisfaction. 45% experienced at least one physical symptom in the previous 4 weeks (change in food habits, difficulty falling asleep and muscle tension). Higher levels of burnout were associated with a more frequent experience of symptoms. Females showed higher levels of emotional exhaustion. Physicians experienced symptoms less frequently than nurses. Provide timely counseling services and support systems to mitigate the massive impact.		III
Wu Y, et al. (2020)^(14)^	Cross-sectional study to compare the frequency of burnout between physicians and nurses working in usual and frontline wards, including 190 participants, 96 of whom worked in the frontline wards.	The frequency of burnout was significantly lower in the frontline group than in the usual wards group in the past 2 months during the COVID-19 pandemic. HCPs in usual wards may have perceived less control over new policies and procedures. HCPs in frontline may have felt closer to the key decision makers and have had access to more timely and accurate information. Much attention is paid to those who work directly with infected patients. 76% of participants from the frontline strongly disagreed or disagreed that he/she felt more burnout now compared with before the COVID-19 pandemic. Participants continuing to work in their usual wards were more worried about themselves or a family member becoming infected.	-	III
Shahin T, et al. (2020)^(15)^	Cross-sectional study to compare anxiety and burnout levels between HCPs working in emergency service with other HCPs in Turkey during the COVID-19 pandemic.	The burnout score of pandemic area and the intensive care unit (ICU) workers were similar to those in the emergency service and ambulance. The emotional score of the emergency and ambulance workers was higher. The burnout score of pandemic fields and ICU workers was significantly higher than radiology, laboratory, and office workers. Burnout was higher in women and HCPs who lived with their relatives >65 years of age. Burnout in doctors and nurses was higher than other HCPs. PPE was associated with higher burnout score.	-	III
Kannampallil TG, et al. (2020)^(18)^	Cross-sectional study to investigate the effects of learner exposure to COVID-19 patients in their clinical roles on their mental health and wellness outcomes in 393 physician trainees (residents and clinical fellows) in the United States.	The exposed group had a higher level of burnout compared to the non-exposed group. Multivariate regression showed that trainees who were exposed to COVID-19 patients reported significantly higher burnout. Normalize feelings of emotional distress and reduce stigma by encouraging discussion of the stressors of clinical work. Provide programs that increase accessibility to mental health services for trainees. Provide childcare options for married trainees at night shifts and long hours duties.		III
Hu D, et al. (2020)^(19)^	Cross-sectional study to evaluate mental health (burnout, anxiety, depression, and fear) and the associated factors among 2,014 frontline nurses who were caring for COVID-19 patients in China.	About half of the nurses reported moderate and high job burnout, 60.5% emotional exhaustion, 42.3% depersonalization, and 60.6% personal accomplishment. Emotional exhaustion was positively correlated with skin lesion and negatively correlated with self-efficacy, resilience, intra-family social support, and extra family social support. Depersonalization was negatively correlated with resilience, intra-family social support, and extra-family social support. Personal accomplishment was positively correlated with self-efficacy, resilience, intra-family social support, and extra-family social support. Improve mental health Build self-efficacy and resilience Provide sufficient social support Ensure frontline work willingness		III
Restauri N and Sheridan AD (2020)^(20)^	A comprehensive study to provide a conceptual paradigm for understanding the relationship between burnout, acute stress disorder, and post-traumatic stress disorder (PTSD); as well as an evidence-based review and recommendations for system-based interventions that may reduce physicians’ stress.	Increased exposure to stress and trauma due to acutely increased workplace stress resulting from the pandemic, combined with underlying baseline burnout, may result in rising rates of PTSD among physicians. Causes of burnout: Lack of job control Excessive workload Prolonged work stress Imbalance between demands and skill set Burnout consequences: Decreased productivity Decreased quality of patient care Decreased patient satisfaction Increased turnover Increased medical error Increased substance abuse Increased depression Increased suicide Disrupted relationships	Organization-directed interventions are more effective in preventing and reducing burnout: Decrease the clinical demand via schedule changes Increase team work Increase job control Increase shared decision making Mindfulness and cognitive behavioral therapy. Support an infrastructure that allows HCPs to work from home to decreases exposure and concerns about infection. Education about burnout via expert panel discussions and accessing mental health to increase awareness and early intervention, and reduce stigma. Increase the sense of safety in the workplace with clear communication from leadership to increase the sense of safety and stability, and increase team work. Improve a culture of psychological safety in the workplace. Individual interventions; such as micropractices (strategies requiring just a few seconds to manage stress).	IV
Luceno-Moreno L, et al. (2020)^(21)^	Cross-sectional study to analyze posttraumatic stress, anxiety, depression, and associations between burnout and resilience in 1,422 Spanish HCPs during the COVID-19 pandemic	Anxiety and depression were positively and significantly related to emotional exhaustion and depersonalization. High scores on emotional exhaustion and depersonalization are risk factors for mental health, with resilience and personal fulfilment being protective variables. Resilience is a protective factor. Promote resiliency		III
Zerbini G, et al. (2020)^(22)^	Cross-sectional study to compare the psychosocial strain in 111 HCPs [75 nurses (45 COVID-19 wards vs. 30 regular wards) and 35 physicians (17 COVID-19 wards vs. 18 regular wards)] during the COVID-19 pandemic.	Participants with increased scores for exhaustion, depression, anxiety, and stress reported a higher fear of being infected. Feeling more stressed at work was associated with burnout. Fear was correlated with higher emotional exhaustion and depersonalization. Nurses working in the COVID-19 wards reported higher levels of exhaustion. Physicians had similar scores regardless of the type of ward. The most common causes for psychosocial burden: Job strain (increased workload, organizational changes in working team, conflicts with colleagues) Uncertainty about the future (healthcare system and economic crisis) Concerns about one’s safety and the safety of the family Family, friends, and leisure time lead to more resilience. Social support was one of the most important resources to cope with the psychological burden following the pandemic. Provide social support Arrange more off-time for spending time with family and friends. Provide psychological support Reduce working hours. Keep working teams stable. Improve communication and recognition. Provide clear and available guidelines.		III
Morgantini LA, et al. (2020)^(23)^	Cross-sectional study to describe the burnout’s contributing factors among 2,707 HCPs (physicians such as residents and fellows; nurses) during the COVID-19 pandemic, from 60 countries.	51% reported burnout (higher than previously reported rates) due to high workload, job stress, and time pressure, and limited organizational support. Burnout was associated with: Work impacting household activities Feeling pushed beyond training Exposure to COVID-19 patients Making life-prioritizing decisions Adequate PPE High-income compared to low- and middle-income countries	Actions from healthcare institutions and other governmental and non-governmental stakeholders, included: Providing additional training and mental health resources Providing updated guidelines Strengthening organizational support for HCPs’ physical and emotional needs Supporting family-related issues (e.g. helping with childcare, transportation, temporary housing, wages) Acquiring PPE Methods focused on mindfulness, stress management and small group discussion.	III
Wan Z, et al. (2020)^(24)^	Cross-sectional study to evaluate the status of burnout and anxiety among 1,011 Chinese nurses working for at least one week during COVID-19 epidemic and the influencing factors.	The predictive factor for emotional exhaustion: 5 years or less working experience Living in hospital dormitory 3 or more night shifts weekly A better level of knowledge of COVID-19 Having confirmed or suspected medical staff with infection around For cynicism: Intermediate tile Personnel agency Working in isolation ward Living in hospital dormitory 3 or more night shifts weekly A level of knowledge of COVID-19 Having confirmed or suspected medical staff with infection around For personal accomplishment: No siblings Living at a hotel 9 or more hours of daily work Level of knowledge of COVID-19 Having confirmed or suspected medical with infection staff around	Perform series of measures to care for HCPs, such as increasing remuneration package, implementing first-line personnel life security, and strengthening personal protection. Increase the knowledge and skills of HCPs who care for COVID-19 patients. Managers pay more attention to the HCPs safety, and take protective measures and care for them.	III

**Table 2.** Articles in the format of opinion, editorial, letter, or prospective

**Author**	**Aim**	**Main findings**	**Recommendations**
Hartzband P and Groopman J (2020)^(6)^	To describe the causes and solutions of burnout in physicians during the COVID-19 pandemic.	Recommendations have targeted the doctor, proposing exercise classes and relaxation techniques, snacks and social hours for decompressing, greater access to child care, hobbies to enrich free time, and ways to increase efficiency and maximize productivity. Intrinsic and extrinsic motivators would have additive or synergistic effects. Other opinions stated that tangible extrinsic motivators, such as monetary rewards, can paradoxically weaken intrinsic motivation. Three pillars support professionals’ intrinsic motivation and psychological wellbeing: autonomy, competence, and relatedness. Physicians and their family and friendships suffer from the electronic health records' demands that invade doctors’ homes and consume the time enjoyed in vital relationships, worsening emotional exhaustion. Give back autonomy, competence, and relatedness to physicians.	
Upadhyay P (2020)^(10)^	To describe the burnout in HCPs in Nepal and its factors and recommendation during the COVID-19 pandemic.	The positive factors for burnout: Long working hours Increasing bureaucratic tasks Continued exposure to human suffering and death Constant need to be compassionate to patients and their family members Increase violence against HCPs and feeling unappreciated Lack of PPE, respirators, and hospital infrastructure to support the increasing hospitalizations Moral and ethical dilemma during decision making process A sense of guilt and regret for the general lack of preparedness to support the patients Fear for one’s life and safety Delay in instituting measures to effectively address the problems Inappropriate work culture Hierarchical structure of the medical fraternity Neglecting physical, mental, social, and emotional wellbeing by HCPs Mandated time away from work (especially for frontline HCPs) Practice mindfulness Assess own physical and mental health by HCPs Train physical, mental and emotional self-care Change in one’s lifestyle Implement self-driven technique Establish a healthier work environment Change work culture	
Fessell D and Cherniss C (2020)^(13)^	To describe micropractice for burnout prevention and emotional wellness during the COVID-19 pandemic.	Institutional and individual interventions for addressing burnout and promoting wellness: Decreased workload, improved work schedules and electronic health record, mindfulness-based stress reduction, and personal coaching. Physicians enjoy highly actionable tools that require minimal time to learn and implement (micropractices). Micropractices only require a few seconds to a few minutes to implement.	Although many structural and cultural changes are needed, micropractice is a suitable strategy to prevent burnout. Suitable times for micropractices: Hand hygiene for self-awareness and self-management. When logging into the electronic health records. Hearing the concerns of family or friends When waiting at a red light Before answering e-mails or texts When brushing teeth Take a moment to name one’s emotions, especially challenging emotions. Write down three good things. Share the personal practices around burnout prevention and wellness in a workshop setting. Do diaphragmatic breathing.
Houtrow AJ (2020)^(25)^	To compare symptom management vs. treating the cause of burnout.	When HCPs cannot act in accordance with the moral obligations to the patients, it may result in psychological distress. Symptom interventions, such as mindfulness training are valuable and important, but a shift to addressing the root causes is definitely essential. HCPs suffer when the public health response is inadequate. Use mindfulness practices, relaxation techniques, exercise Promote clinician well-being	
Shah K, et. Al (2020)^(26)^	To describe measures to address the physicians' burnout during the COVID-19 pandemic.	Physicians, residents, fellows, and other HCPs experience a varying degree of burnout. Physician burnout factors: Work factors: high workloads and prolonged work hours Personal characteristics: work-life imbalance, inadequate support, sleep deprivation Organization factors: workload expectations, insufficient rewards, and interpersonal communication negative leadership Other factors: lack of control over procedures, infection control measures, the false notion of safety precautions, poor communication and directives, lack of preparedness and emotional support, inadequate PPE, and perceived fatality	Empower physicians by providing essential resources adequately (PPE, beds, medicines, ventilators, educational guidelines, and research updates). Provide consistent and updated guidelines regularly to staff for managing patients through triage based on the case priority and severity. Recruit additional HCPs and administrative staff Facilitate the setup of telemedicine and telepsychiatry services. Provide support with clear communication from the leadership regarding quarantine directives, guidelines, and management protocol. Restrict excessive workload by scheduling breaks and limiting work hours in emergency and intensive care units. Provide regular psychosocial support, essential basic needs, mindfulness sessions, and resilience training. Daily screening of vital signs, possible symptoms of infection, and signs of burnout. Train the expertise of the residents and fellows as a frontline worker to handle patients. Protect and support residents and fellows by creating an action plan and temporarily deferring the rules for training and board eligibility.
Nadler B, et al. (2020)^(27)^	To describe the strategies for supporting oncology HCPs during the COVID-19 pandemic.	The wellness of HCPs is a spectrum, from engagement to burnout; that individual characteristics, experiences and organizational factors can influence one’s position on this spectrum.	Interventions to decrease burnout: Organizational level: 5 principles (“hear me,” “protect me”, “prepare me”, support me” and “care for me”). “CREATE” (Compassion and Resilience Team-building): pairs a psychosocial services professional with clinical managers to offer support and implant low-dose interventions into clinical teams using a coaching and psychological first aid model. A toolkit with information on accommodation, grocery delivery, safety, coping and mental health resources.
Janeway D (2020)^(28)^	To describe the role of psychiatry in treating burnout among nurses during the COVID-19 pandemic	Burnout is related to: Lack of health insurance Lack of resiliency Poor communication skill Lack of safe environment to express their anxieties, fears, grief, and hopeless/helpless feelings Poor self-care skills	Using psychiatrists and mental health professionals (mental health services) Consultation liaison (CL) psychiatry provide assistance through liaison meetings, stress management programs, and curbside consults to help reduce the risk of burnout. CL provide a safe environment for HCPs to express their anxieties, fears, grief, and hopeless/helpless feelings in addressing the mental health needs of their patients. Journal club meetings Providing better communication skills Stress management programs (one or two sessions, an 8-12- week program or open weekly sessions) Improve relaxation skill Resiliency training program Improving cognitive, behavioral, self-care skills, yoga, tai chi Grief counseling Brainstorming around ways to change workplace and workload Building workload and organizational management skills Music and art therapy Writing workshops
Ong AM (2020)^(29)^	To describe the impact of the COVID-19 pandemic on medical education and resident burnout in a postgraduate program.	Burnout risk factors among residents: Separation from their colleagues and families Loss of autonomy Disruption of training and reduction the usual cases and procedures Residents mentioned fear for their own health as they were in the frontline.	Plan a 24-hour hotline with a psychologist and weekly mindfulness sessions over video conference. Create a clear and open channel of communication between the program director and the residents. Communicating with their colleagues over social media or email frequently. Implement a ‘no questions asked’ policy in the event of any resident taking sick leave.
Ong AM (2020)^(30)^	To describe burnout in a GI fellowship program during the COVID-19 pandemic.	The cause of burnout: Reduction in elective procedures Concern about the training program Concern about maintaining their procedural skills, due to deployed on isolation wards Worry about losing procedural and clinical competence and job uncertainty Fear for their own health and well-being due to caring for large numbers of COVID-19 patients Increase in overall working hours due to shortage of staff Long time self-isolation periods away from their families and colleagues Decrease in the social interactions between families and colleagues Loss of autonomy	Change the assessment method of competencies Provide supplementary teaching programs for residents missing out training programs Create social media chat group for communications Cancel the formal presentations and teaching programs to allow more time to spend with families Faculty stepping in to relieve residents of clinical workload Arrange weekly mindfulness sessions Availability of 24-h hospital psychologist Clear communication by program leadership regarding continuation of training and implications on job prospects
Sasangohar F, et al. (2020)^(31)^	To describe lessons learned from a high-volume intensive care unit where the frontline HCPs work, about burnout and fatigue during the COVID-19 pandemic.	Frontline HCPs emotionally breaking down, due to the added pressure to choose between family responsibilities and their inner sense of duty toward patients. It was seen support from medical leadership, public and private acknowledgments, community support (food sent to care units), music therapy, counseling services, chaplain services, and accommodations in work schedules. Organizational adaptations: allocation of more resources (float nurses, physicians, patient care assistants, and new equipment) New protocols were published in response to the pandemic which were perceived as complex and premature. Policy overload coupled with mismatching policy from different levels or sources Each subspecialty follows guidelines provided by their respective professional societies for various procedures. New policies were developed by the hospital. Social distancing and quarantine protocols resulted in unprecedented overall societal stress and anxiety. Job insecurity and uncertainty about future occupational stability increased for some specialists like some private anesthesia groups due to canceling and delay in routine elective surgeries. Organizational adaptation: Rapidly assembled the incident command team. Responsiveness and constancy of leadership–employee communication Adaptation of human resources policies to employee needs. Using digital communication tools for remote work and intra institutional collaborative efforts. Communications between specialist and learning through popular social media platforms. Opportunity for innovations and adoption of alternative care delivery methods like telemedicine and virtual ICUs.	Develop guidelines to increase teamwork between different specialists and decrease confusion and frustration. Support increased demand for disinfectants, cleaning supplies, PPE, and other medical equipment for health care and community use. Assess updated information about availability of testing kits and PPE for to reduce the anxiety associated with uncertainty, and reduce unproductive information seeking and emotional stress. Use daily rounds along with communication technologies to access reliable information sources. Provide structured training on large-scale disaster management and response. Improve innovation as well as provide technical oversight to ensure that new designs meet minimum safety requirements. Employ other well-trained resource of medical professionals in the form of internationally trained physicians, nurses, medical technicians, and other HCPs. Provide wearable sensors for noninvasive monitoring of fatigue, stress, and sleep biomarkers for timely intervention. Use mobile health (mHealth) tools for facilitating the mental health self-management. Use simple methods such as breathing exercises, biofeedback, and mindfulness to reduce cute episodes of stress and anxiety. Use telehealth services to enable peer-support and occupational counseling.
Sultana A, et al. (2020)^(32)^	To describe challenges and evidence-based interventions for burnout among HCPs during COVID-19 pandemic.	Psychological stressors for burnout: Working hard during emergencies or stressful conditions Workload Sleep deprivation Depression Lack of resilience Poor self-management Inappropriate work schedule Inappropriate workflow management Poor communications skills Poor coping skill Unsafe workplace Lack of mental health services Increase potential burnout awareness: can reduce stigma towards mental health conditions and develop resiliency. Decrease the workload Improve work schedule Promote self-management Initiate mindfulness-based stress reduction Mental health promotion activities Provide mental health services Involve mental health experts in multidisciplinary COVID-19 teams Hold group-based counseling or peer-support sessions Balance use of electronic health records Monitor healthy work conditions Address the risks of workload and workplace stress Deliver mental health services through digital platform Improve workflow management Enhance interoperability Arrange discussion and exchanging opinions Improve communication skills Provisos for adequate rest and exercise Organize workshops on coping skills Devise policies and practices Develop supportive work environment	

**Table 3:** The summary of recommendations for preventing or reducing burnout among healthcare providers (HCPs) of COVID-19 wards

**The society**	**Workplace conditions and organizational behavior**	**Digital technologies**	**Mental health status**	**Personal characteristics**
Provide social support Increase social interactions Decrease social violence	Promote work culture Relieve workplace stressors Develop healthier and supportive work environment Give back autonomy, competence, and relatedness to physicians Improve workflow management Organize services with an emphasis on reducing workload (improve work schedule, reduce working hours, schedule breaks, floating work schedule, limit work hours) Improve communication skills Hold workshops on coping skills Arrange discussion meetings Increase interoperability Brainstorming around ways to change workplace and workload Increase shared decision making Provide the opportunity for having adequate rest and exercise Increase teamwork and job control Develop policies and methods to reduce burnout Support an infrastructure that allows HCPs to work from home Provide cross-sectoral and inter-organizational collaboration to share information, resources, support Use strategic distribution of human resources Increase the number of human resources by hiring more HCPs Employ foreign HCPs Decrease clinicians from nonclinical tasks and medical notes Daily screening of vital signs, possible symptoms of infection, and signs of burnout Develop clear and updated guidelines and protocols for different situations Develop practical training about protective interventions Provide essential resources adequately (PPE, beds, medicines, ventilators) Provide mental health services and increase the empowerment of HCPs through it Balance use of electronic health records Talk about concerns with colleagues and friends through web-based social media Use digital communication and social media platforms Use it for training Use it for sharing information Use it for immediate access to valid and up to date information Use it for virtual support groups such as book club, journal club, or coffee talk, virtual dinner, and happy hours Use telehealth services to enable peer-support and occupational counseling. Use it in the format of telemedicine and virtual ICUs Provide wearable sensors for noninvasive monitoring of fatigue, stress, and sleep biomarkers for timely intervention. Use it as mobile health tools (mHealth)		Provide mental health services Provide counseling and support systems Involve mental health experts in multidisciplinary COVID-19 teams Use consultation liaisons Promote mental health Promote resiliency Promote self-management Start mindfulness-based stress control activities Teach physical, mental, and emotional self-cares Improve relaxation skill Arrange stress management programs Use methods focused on mindfulness, stress management and small group discussion Provide cognitive behavioral therapy, yoga, tai chi, grief counseling, and music and art therapy Provide writing workshops through mental health services Increase health literacy (happiness, exercise, drinking water, being joyful, and having a good sleep) Include periods of rest and relaxation in shift program and schedules Provide restroom and possibility of taking a shower in the workplace Mandatory time away from work for spending with family, friends, hobbies, and rest Social support within the family Interaction with family members and loved ones Support family-related issues especially in married women (e.g. helping with childcare, transportation, temporary housing, and wages)	

## Limitations:

The main limitation of this systematic review stems from the lack of high-quality evidence and interventional studies. No study surveyed any intervention to prevent or reduce burnout, and the provided recommendations were based on the authors' experiences and opinions. None of the studies followed up the participants, and all assessments were done according to the participants’ self-reporting and declaration. No study established a causal relationship because of their methodology. 

## Conclusion:

Awareness of healthcare managers and policymakers from burnout among HCPs, who are working at COVID-19 wards, and administration of appropriate solutions to prevent or reduce the burnout are necessary. Paying attention to the mental health issues, reducing the workload of HCPs through adjusting their work shifts, reducing job-related stressors, and creating a healthy work environment may prevent or reduce burnout. Future, large and multicenter studies on HCPs of COVID-19 wards are necessary to identify the frequency, associated factors, and effective preventative strategies of this phenomenon.

## Implications of key findings

The available early-stage and low-quality evidence cannot provide convincing support in favor of or against a particular recommendation to prevent or reduce burnout in HCPs of COVID-19 wards. This is mainly because of the heterogeneity with respect to the participants and applied tools, different suggestions, absence of any intervention, and not following the participants.

However, the results of this study showed that the policymakers can take measures to prevent or reduce burnout in the five introduced areas. However, more large and interventional studies are highly recommended to identify effective solutions and measure their effectiveness.

## Standard Protocol Approvals, Registrations, and Patient Consents

The Shiraz University of Medical Sciences Institutional Review Board approved this study and systematic review (IR.sums.med.rec.1399.322).

## Systematic review registration number

The review protocol was not previously registered.

## Availability of data and material

Data sharing is not applicable to this article.

## Ethical issues

This study was approved by the vice-chancellor of research and technology (Grant No. 23376), as well as the local Ethics Committee (IR.sums.med.rec.1399.322) of Shiraz University of Medical Sciences.
